# Tibia length is an appropriate standard for evaluating hypertrophy in streptozotocin-induced diabetic complications

**DOI:** 10.1007/s00210-025-04413-3

**Published:** 2025-07-17

**Authors:** Miao Qin, Feng Shao, Teresa Fernandez, Yixin Wang, Thomas Wieland, Christiane Vettel, Yuxi Feng

**Affiliations:** 1 Experimental Pharmacology Mannheim, European Center for Angioscience, Medical Faculty Mannheim, Heidelberg University, Ludolf-Krehl-Str. 13-17, 68167 Mannheim, Germany; 2 DZHK (German Center for Cardiovascular Research), Partner Site Heidelberg/Mannheim, 68167 Mannheim, Germany

**Keywords:** C57 BL/6 substrain, Tibias, Body weight, Diabetic complications, Hypertrophy, Standard

## Abstract

The pathogenesis of diabetic complications has been extensively studied in streptozotocin (STZ)-induced diabetic mice. Body weight (BW) is still used to standardize organ hypertrophy, but it may be unreliable due to significant weight loss in this model. This study aimed to assess the reliability of using tibia length (TL) as a common standard for organ hypertrophy in emaciated diabetic mice. Diabetes was induced by STZ in wild type mice with C57BL6/N (6N) and BL6/J (6J) background. TL and the weights of the heart (HW), kidneys (KW), and bladder (BLW) were estimated at 1-, 3-, and 6-month post-diabetes induction. The 6N mice were more sensitive to STZ than the 6J mice. In 1-month diabetic mice, the HW, HW/TL, and HW/TL^3^ ratios decreased in both substrains. In contrast, BLW/TL and BLW/TL^3^ ratios increased in the 6J but not in the 6N mice, suggesting that the 6J is more suitable for early-stage diabetic bladder research. In 3-month diabetic mice, KW/TL and KW/TL^3^ ratios significantly increased in the 6J mice, suggesting that the 6J strain is more appropriate for studying early-stage diabetic nephropathy. In 6-month diabetic mice, both substrains were comparable. Interestingly, in non-diabetic 6-month 6N mice, HW, KW, and TL were positively correlated with BW, while in non-diabetic 6J mice, TL and TL^3^ were consistently correlated positively with HW. TL is a more reliable metric for indexing organ hypertrophy in emaciated diabetic mice. Additionally, the C57BL/6J substrain is more appropriate than C57BL/6N for studying early-stage diabetic nephropathy and cystopathy.

**Supplementary Information:**

The online version contains supplementary material available at 10.1007/s00210-025-04413-3.

## Introduction

Diabetes is a chronic metabolic disorder characterized by elevated blood glucose (BG) levels, which can result in both acute and chronic complications, ultimately leading to severe vision loss, heart failure, and renal dysfunction in the advanced stages. The report of the International Diabetes Federation in 2021 underscores a disconcerting trend that the prevalence and incidence of diabetes are rising rapidly worldwide. As the population of individuals diagnosed with diabetes continues to expand, there is also a significant uptick in associated organ complications, which creates a substantial financial challenge for healthcare systems globally (Sun et al. 2022; R. Williams et al. 2020a, b).

Diabetic cardiomyopathy is defined as ventricular dysfunction in diabetic patients in the absence of coronary artery disease and hypertension (Dillmann 2019; Rydén et al. 2013). It is characterized by structural and functional abnormalities, including left ventricular hypertrophy and diastolic dysfunction, which can progress to heart failure with preserved ejection fraction, and eventually lead to systolic dysfunction accompanied by heart failure with reduced ejection fraction (Jia et al. 2016; Marso et al. 2016). Diabetic nephropathy, a microvascular complication of diabetes, manifests as persistent albuminuria and a progressive decline in renal function. Its pathological characteristics include thickening of the glomerular basement membrane, mesangial cell expansion, renal hypertrophy, and fibrosis, all of which are triggered by biochemical alterations under hyperglycemia (Thomas et al. 2015). Additionally, diabetes also induces damages to the nerves of the urinary tract, leading to bladder dysfunction defined as diabetic bladder, which includes impaired bladder contractility, voiding dysfunction, and overactive bladder syndrome, with or without urinary incontinence (Brown et al. 2005; Michel & Barendrecht 2008). In various rodent models that are utilized for studying diabetes, it is noted that there is a significant increase in the size of the bladder, a pathological condition known as bladder hypertrophy (Arioglu Inan et al. 2018; Ellenbroek et al. 2018; Erdogan et al. 2023).

Most knowledge about the pathogenic mechanisms of diabetic complications has been derived from rodent models of diabetes. Streptozotocin (STZ) is a commonly used chemical for inducing diabetes and its complications in mice. Intraperitoneal STZ injections can ablate pancreatic beta cells, leading to reduced insulin levels, and consequently elevated BG and hemoglobin A1C (HbA1c) levels. Tissue hypertrophy is an early feature of diabetic complications, occurring in the diabetic heart, kidney, and bladder, where resident cells increase in size. Body weight (BW) has traditionally been used to normalize organ hypertrophy during the development and progression of diabetic complications (Adhikary et al. 2004; Nemoto et al. 2006; Yang et al. 2019). In the heart, some validated techniques can reliably assess cardiac hypertrophy and function in diabetic mouse models (Bugger & Abel 2009; Jasińska-Stroschein 2023). Left ventricular hypertrophy has been quantified using the heart weight (HW)-to-BW ratios and ventricular anterior/posterior wall thickness measurements obtained via echocardiography (D'Souza et al. 2011; Nemoto et al. 2006). However, STZ-induced diabetic mice often experience significant weight loss, which makes normalization by BW unreliable (Goyal et al. 2016). In diabetic research, tibia length (TL) is emerging as a standard reference for normalization. TL remains constant after maturity and serves as a more reliable parameter for standardization in STZ-induced diabetic models (Hagdorn et al. 2019). Yin et al. compared HW normalized by TL versus BW in 5- to 28-month-old male Wistar rats, finding that left ventricles underwent 17% hypertrophy when normalized by TL versus 38% when normalized by BW (Yin et al. 1982). Histological measurements indicated approximately 15% hypertrophy, more closely aligning with the estimates based on TL than those based on BW. As a result, the application of HW normalized to TL has become a standard for normalizing cardiac hypertrophy (Cao et al. 2015; De Blasio et al. 2020; Tate et al. 2019). Nevertheless, to the best of our knowledge, only a few studies have quantified hypertrophy in the diabetic kidney by normalizing the KW to TL (Bivona et al. 2011; Thibodeau et al. 2014; Uehara-Watanabe et al. 2022). Standardizing organ hypertrophy in STZ-induced diabetic complications remains a challenge.

This study aimed to evaluate whether TL can serve as a reliable standard for normalizing the quantification of organ hypertrophy in diabetic complications, such as hypertrophy of the kidney and bladder in STZ-induced diabetes model.

## Materials and methods

### Animals

Approval for the mice used in this study was granted by the local ethics committee (Regierungspräsidium Karlsruhe, Germany). All animal experiments were conducted in accordance with institutional guidelines. The male mice used in this study were control mice from other studies and were second or third-generation after regular cross-breeding back to the C57BL6/N (6N) or C57BL6/J (6J) background. Two to three non-diabetic and diabetic mice were housed in a medium-sized cage. The mice were kept at a constant temperature of 21 °C with 55% relative humidity. Illumination was maintained at a light intensity of 150 lx with a 12-h day/night cycle. Mice had free access to food and drinking water. The standard pelleted diet consisted of 9% fat, 33% protein, and 58% carbohydrates. The powdered feed of the same composition mixed with water was also provided every 2 days to both diabetic and non-diabetic mice to improve palatability and accessibility, ensuring hydration, adequate nutrient intake, and animal welfare in diabetic mice, as well as supporting weight maintenance.

### Induction of diabetes

STZ (Sigma-Aldrich, USA) for the induction of type 1 diabetes was freshly dissolved in citrate buffer (pH 4.5). After a 4-h fasting period, the 2.5-to-3-month-old mice were injected intraperitoneally with STZ (145 mg/kg BW) as described in the previous studies (Furman 2021; Hammes et al. 2004; Pfister et al. 2008). Following the injection, the mice were given 5% glucose in their drinking water for 24 h. BG measurements were performed using a drop of blood from the lateral tail vein, measured with a glucometer with a maximum detectable BG level of 600 mg/dL. BG levels were monitored on the third day after diabetes induction, and levels exceeding 250 mg/dL indicated successful diabetes induction.

### Measurement of general parameters

BW was measured before STZ injection, weekly during the first month, and then monthly after diabetes induction and at sacrifice. After the diabetes model was established, BG levels of diabetic animals were monitored weekly for the first month, then monthly, and at the end of the experiments. Since non-diabetic mice did not exhibit significant changes in BG or BW as rapidly as diabetic mice, in line with the 3Rs principle (Replacement, Reduction, and Refinement) in animal studies, we reduced the frequency of BG measurements in non-diabetic mice to once per month to minimize unnecessary stress. Mice in the 3-month and 6-month diabetic groups received individualized subcutaneous insulin injections (1–2 units of glargine, Sanofi, Germany) three times a week began no earlier than 2 weeks post-STZ injection, allowing BG to remain high but matching models of chronic, poorly controlled diabetes while maintaining the better general condition and prevent ketoacidosis in the diabetic mice (Hu et al. 2022; Klemis et al. 2017; Teuma et al. 2022).

### Organ and tissue collection

Mice were euthanized according to institutional guidelines at 1, 3, and 6 months after diabetes induction. According to our earlier study on diabetic vascular damage, vascular morphological changes begin around 3 months (e.g., pericyte loss) and progress markedly by 6 months (e.g., formation of acellular capillaries) in the retina, while no visible vascular structural alterations are seen at 1-month post-diabetes induction (Chatterjee et al. 2020; Hammes et al. 2004). Since endothelial cells are distributed throughout the body, similar microvascular alterations likely occur in other organs. Therefore, 1-, 3-, and 6-month time points were used to align with previous work on diabetic complications. BW at the time of sacrifice was recorded. The mice were anesthetized using isoflurane (1214, CP-pharma, Switzerland), followed by cervical dislocation. BG and HbA1c levels were measured, and HbA1c measurement was performed with an HbA1c analyzer according to the manufacturer’s manual (Infopia Clover A1c Analyser, EuroMedix, Germany). The heart, kidneys, bladder, and tibias were collected. The heart was immediately placed in physiological saline solution, and blood in the heart was expelled by automatic cardiac contraction and dilatation. Residual fluid was removed by rolling the heart on a soft tissue paper before weighing. The bladder was similarly emptied by rolling. After dissection of the tibias, muscles and residual tissues surrounding the tibia were removed, and the TL was measured using a digital vernier caliper (Alpha Tools Digitaler Messschieber, Alpha Tools, Germany). The weights of organs were measured using a scale with 0.1 mg precision.

### Statistical analysis

All statistical analyses were performed using GraphPad Prism 5 (GraphPad Software, La Jolla, CA, USA). Data are presented as Mean ± SD. The organ weights and the ratios of HW, bladder weight (BLW), and kidney weight (KW) compared to BW, TL, or cubic tibia length (TL^3^) were calculated. Statistical significance was assessed using Student’s *t*-test or analysis of variance. Correlations between organ weights and standards were evaluated using Pearson correlation analysis and simple linear regression. *P* values < 0.05 were considered statistically significant.

## Results

### Differential sensitivity of C57BL/6 substrains to streptozotocin

**Fig. 1:**
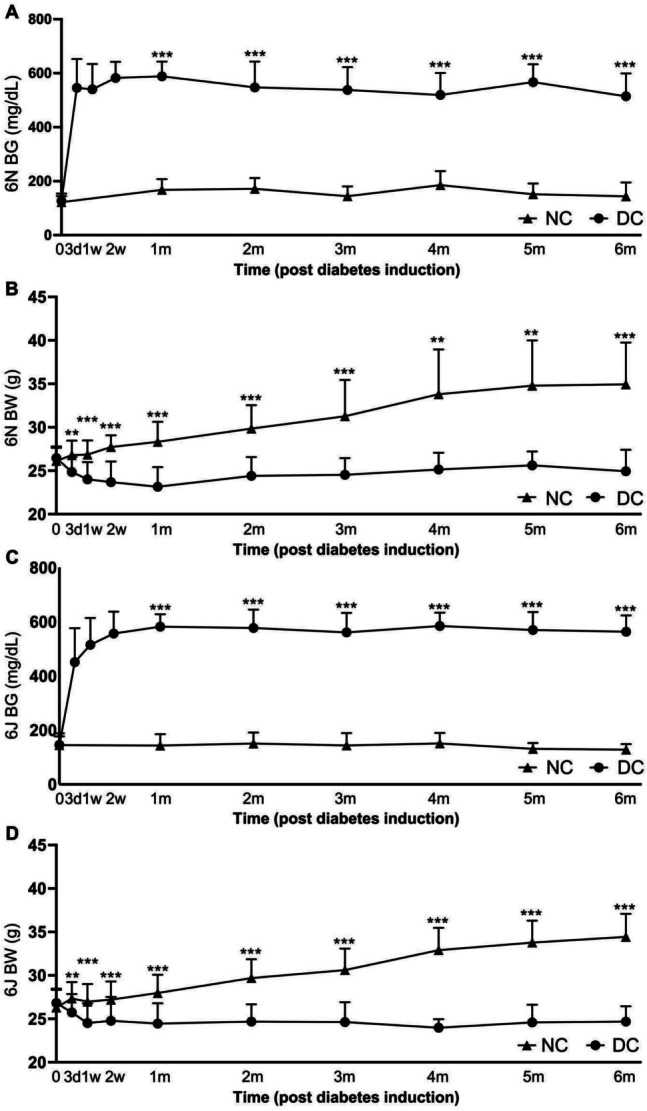
The 6N mice are more sensitive to diabetes induction than the 6J. The random blood glucose (BG) levels and body weight (BW) were measured at 3 days (3d), 1 and 2 week(s) (w), and then monthly (m) after STZ injection. **A** and **C** BG levels of non-diabetic (NC) and diabetic (DC) 6N and 6J mice. **B** and **D** BW of NC and DC 6N and 6J DC mice. *n* = 6–35. Unpaired *t*-test. *p < 0.05, **p < 0.01, ***p < 0.001

The BW and random BG levels of mice with 6N and 6J background were monitored over the experimental periods. In both 6N and 6J substrains, hyperglycemia was successfully induced by STZ using a protocol of induction. BG levels in both substrains peaked at the end of the first month post-STZ injection, and remained elevated for subsequent time periods (Fig. 1A and C). Despite using the same diabetes induction protocol, the BG response within the first month differed between the two substrains. BG levels in 6N mice rapidly approached 600 mg/dL by day 3 post-diabetes induction (affecting 76% mice). In contrast, only 35.71% 6J mice reached this level simultaneously. Statistical analysis of BG at 3 days in both strains showed that 6N mice seemed to be more sensitive than 6J mice to STZ induction (ANOVA, *p* < 0.01).

In non-diabetic mice, BW consistently increased by approximately 33.79% in 6N mice and by 30.88% in 6J mice at the end of the six months (Fig. 1B and D). The diabetic mice, however, showed significant BW loss within the first month post-diabetes induction, with a decline of 12.67% in 6N and 8.82% in 6J mice. BW in diabetic mice stabilized for the remaining study period. Diabetic mice with BG levels above 600 mg/dL and over 10% BW loss received insulin supplementation starting from one month after diabetes induction, which helped stabilize their BW and overall health.

### TL or TL^3^ might be a metric for studying hypertrophy in diabetic cardiomyopathy and cystopathy in the early stage

The diabetic mice were euthanized at three time points: 1-, 3-, and 6-month post-diabetes induction. HbA1c, a parameter indicating long-term BG levels, was significantly elevated in 1-month diabetic mice compared to non-diabetic controls in both 6N and 6J substrains (6N, non-diabetic group 4.75 ± 0.32, diabetic group 9.17 ± 0.69, *p* < 0.001; 6J, non-diabetic group 4.83 ± 0.34, diabetic group 8.36 ± 0.81, *p* < 0.001). Along with increased BG, this indicates successful induction of diabetes and persistent hyperglycemia across all diabetic animals.

**Fig. 2:**
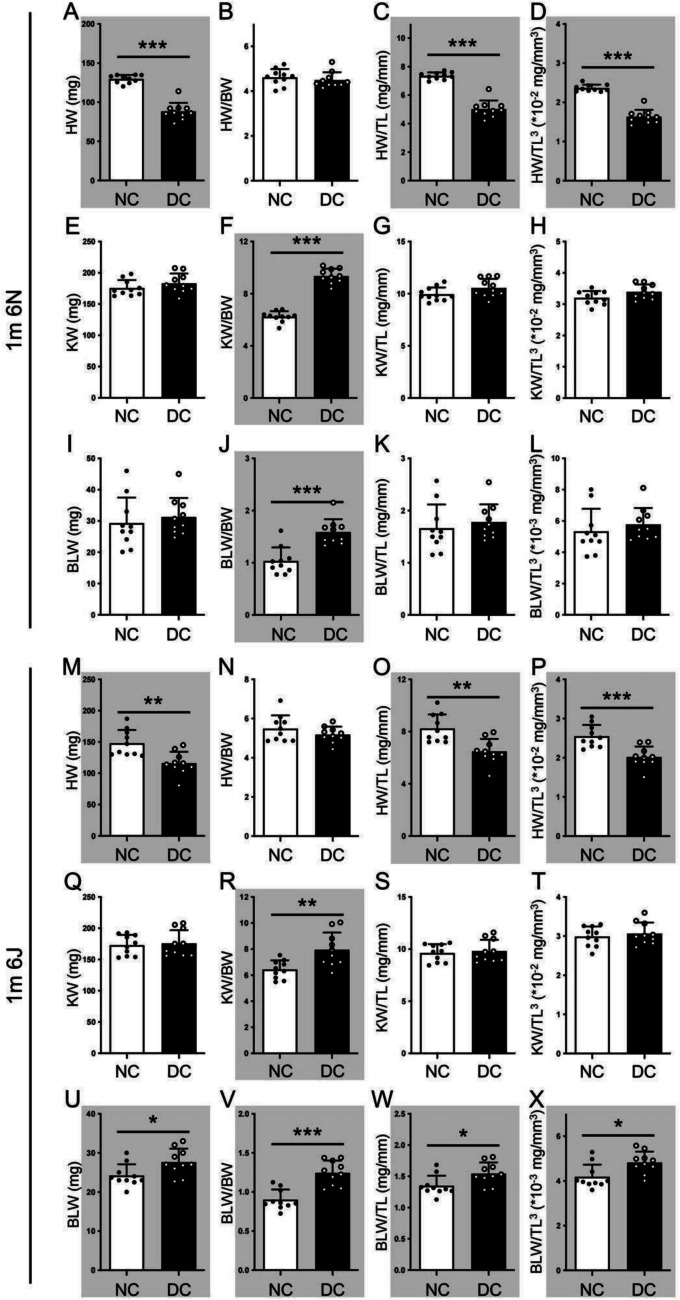
Tibia length and cubed tibia length are more appropriate for assessing diabetic heart and bladder hypertrophy at the early stage. The organ hypertrophy in mice at 1-month post-diabetes induction was assessed by absolute organ weight (mg), organ weight/body weight (BW) ratio, organ weight/tibia length (TL, mg/mm) ratio, and organ weight/cubic tibia length (TL^3^, ⋅10^−2 or−3^ mg/mm^3^) ratio. White column, non-diabetic (NC) mice; black column, diabetic (DC) mice. **A**–**D, M**–**P** the absolute heart weight (HW), HW to BW ratio, HW to TL ratio, and HW to TL^3^ of 1-month DC 6N and 6J mice. **E**–**H**, **Q**–**T** the absolute kidney weight (KW), KW to BW ratio, KW to TL ratio, and KW to TL^3^ of 1-month diabetic 6N and 6J mice. **I**–**L**, **U**–**X** the absolute bladder weight (BLW), BLW to BW ratio, BLW to TL ratio, and BLW to TL^3^ of 1-month DC 6N and 6J mice. Unpaired *t*-test. *p < 0.05, **p < 0.01, ***p < 0.001. m, month(s). Grey background indicates significant alteration between groups

In the 1-month diabetic groups, STZ-induced diabetes led to a reduction in absolute HW by 31.99% in 6N mice (Fig. 2A, *p* < 0.001) and 21.57% in 6J mice (Fig. 2M, *p* < 0.01) compared to non-diabetic animals. However, no significant difference in absolute KW was observed between diabetic and non-diabetic mice in either substrain. Significant elevation of BLW was observed in diabetic 6J animals (Fig. 2U, *p* < 0.05). When normalized to BW, KW/BW and BLW/BW ratios increased significantly in both diabetic 6N mice (Fig. 2F and J; KW/BW, *p* < 0.001, BLW/BW, *p* < 0.001) and diabetic 6J mice (Fig. 2R and V; KW/BW, *p* < 0.01, BLW/BW, *p* < 0.001). Notably, no changes were found in HW/BW ratios between diabetic and control groups in either substrain, suggesting that organ weight-to-BW ratios may yield inaccurate conclusions due to significant BW loss in all STZ-induced diabetic mice.

TL was comparable between diabetic and control mice in both 6N and 6J substrains at 1-, 3-, and 6-month post-diabetes induction (Supplementary Table 1). No significant differences were observed between diabetic and non-diabetic groups, between time points, or between substrains at the same time points. Since TL is unaffected by diabetes induction, organ weight was further standardized to TL. HW/TL ratios were significantly reduced in diabetic mice compared to non-diabetic animals, due to smaller hearts in both substrains (Fig. 2C and O). KW/TL ratios were comparable between diabetic and control mice in both substrains (Fig. 2G and S). BLW/TL ratio increased significantly in 1-month diabetic 6J mice (Fig. 2W) but not 6N mice (Fig. 2K). As suggested by Quint et al., based on mathematical reasoning, organ weight should be considered a three-dimensional parameter, whereas TL is a one-dimensional measure. Therefore, to avoid “pseudo-indexing,” normalization should be performed using TL^3^ rather than TL (Hagdorn et al. 2019). When using TL^3^ for normalization, similar results were seen with organ/TL ratios. Comparing the rising rates of organ weight/standards in figures, the elevation in absolute weights of kidneys and bladder was similar to those standardized to TL and TL^3^, and ratios normalizing to BW showed the most increased rates in all the organs, magnifying the hypertrophic effect. This suggests that TL and TL^3^ are accurate standards for normalizing HW and BLW in 6J mice. The data indicate that the 6J, compared to 6N, is a suitable mouse model for studying early-stage diabetic cystopathy.

### The 6J substrain is more appropriate for studying early-stage diabetic complications than the 6N

**Fig. 3:**
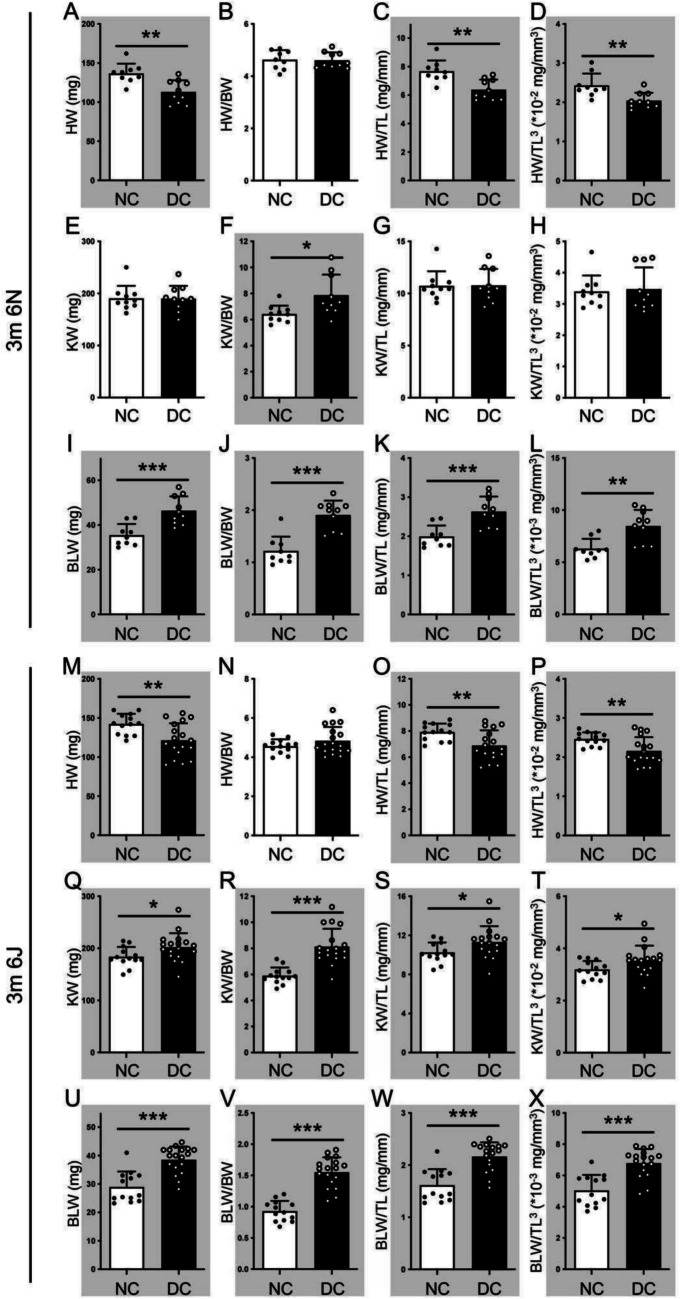
The 6J mice were more appropriate for studying early-stage diabetic complications than the 6N. The organ hypertrophy in mice at 3 months of post-diabetes induction was assessed by absolute organ weight (mg), organ weight/body weight (BW) ratio, organ weight/tibia length (TL mg/mm) ratio, and organ weight/cubic tibia length (TL^3^, ⋅10^−2 or−3^ mg/mm^3^) ratio. White column, non-diabetic (NC) mice; black column: diabetic (DC) mice. **A**–**D**, **M**–**P** the absolute heart weight (HW), HW to BW ratio, HW to TL ratio, and HW to TL^3^ of 3-month DC 6N and 6J mice. **E**–**H**, **Q**–**T** the absolute kidney weight (KW), KW to BW ratio, KW to TL ratio, and KW to TL^3^ of 3-month diabetic 6N and 6J mice. **I**–**L**, **U**–**X** the absolute bladder weight (BLW), BLW to BW ratio, BLW to TL ratio, and BLW to TL^3^ of 3-month DC 6N and 6J mice. Unpaired *t*-test. *p < 0.05, **p < 0.01, ***p < 0.001. m, month(s). Grey background indicates significant alteration between groups

At 3-month post-diabetes induction, HbA1c increased along with continued BW loss in diabetic 6N mice (non-diabetic group 5.30 ± 0.56, diabetic group 9.76 ± 2.07, *p* < 0.05) and 6J (non-diabetic group 4.85 ± 0.61, diabetic group 10.54 ± 1.35, *p* < 0.001). In 6N mice, 3-month diabetic animals showed a significant decrease in absolute HW and an increase in absolute BLW (Fig. 3A and I), with no change in absolute KW (Fig. 3E). The HW/BW ratio did not change (Fig. 3B), but the KW/BW and BLW/BW ratios were significantly elevated (Fig. 3F and J). HW/TL, HW/TL^3^, BLW/TL, and BLW/TL^3^ ratios were significantly changed under hyperglycemic conditions (Fig. 3C, D, K, and L), whereas KW/TL and KW/TL^3^ ratios did not differ between diabetic and control animals (Fig. 3G and H).

In 6J mice, 3-month diabetic animals exhibited significant increases in absolute KW and BLW (Fig. 3Q and U), while absolute HW decreased (Fig. 3M). KW/BW and BLW/BW ratios were elevated in diabetic mice compared to controls (Fig. 3R and V), while HW/BW ratios were comparable between groups (Fig. 3N). All ratios of KW and BLW to either TL or TL^3^ were significantly higher in diabetic mice (Fig. 3S, T, W, and X), while HW/TL and HW/TL^3^ ratios were lower than in non-diabetic mice (Fig. 3O and P). The development of diabetic complications requires weeks to months. Thus, STZ-induced diabetic mice at 1–3 months represent early-stage complications, while the 6-month stage reflects a more advanced or long-term stage. Thus, these findings suggest that 6J mice are more sensitive to hyperglycemia in this mouse model for the development of diabetic complications, especially in the bladder and kidney, and are appropriate models for studying diabetes-related early-stage organ changes.

### Both STZ-induced diabetic 6N and 6J mice are suitable for studies of long-term diabetic complications

**Fig. 4:**
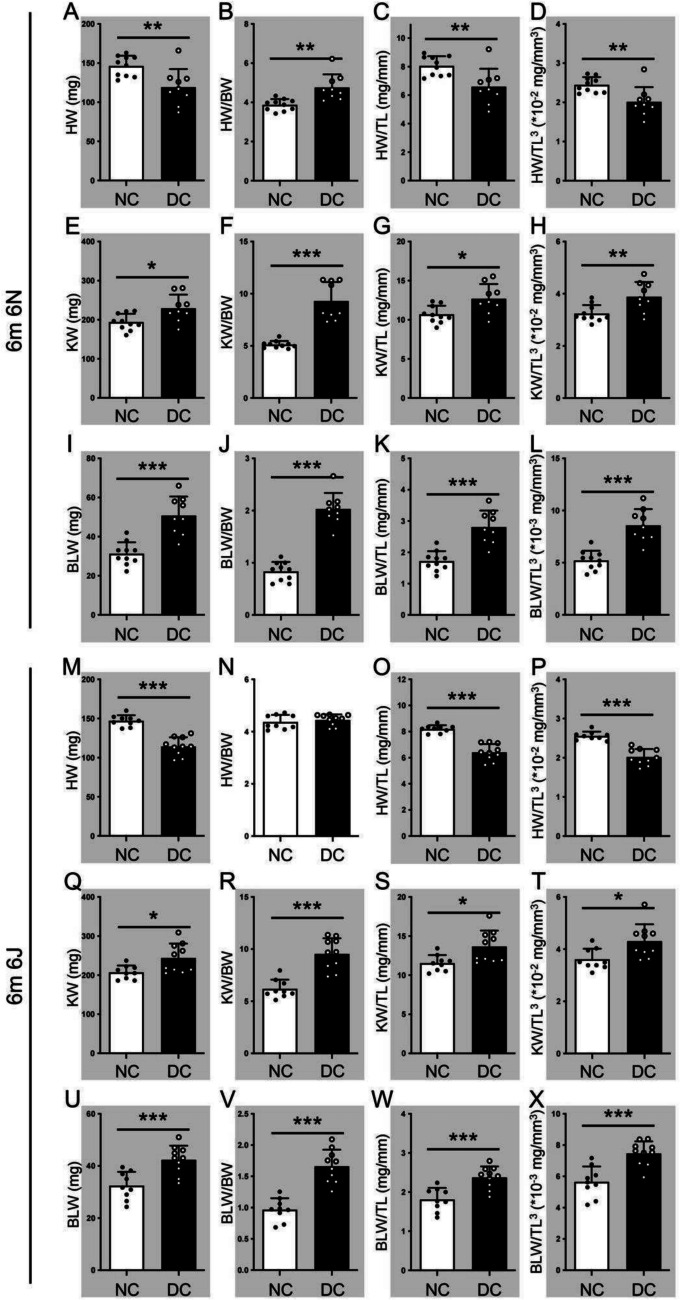
Both STZ-induced diabetic 6N and 6J mice are suitable for studies of long-term diabetic complications. The organ hypertrophy in mice at 6 months of post-diabetes induction was assessed by absolute organ weight (mg), organ weight/body weight (BW) ratio, organ weight/tibia length (TL, mg/mm) ratio, and organ weight/cubic tibia length (TL^3^ ⋅10^−2 or−3^ mg/mm^3^) ratio. White column, non-diabetic (NC) mice; black column, diabetic (DC) mice. **A**–**D**, **M**–**P** the absolute heart weight (HW), HW to BW ratio, HW to TL ratio, and HW to TL^3^ ratio of 6-month DC 6N and 6J mice. **E**–**H**, **Q**–**T** the absolute kidney weight (KW), KW to BW ratio, KW to TL ratio, and KW to TL^3^ ratio of 6-month diabetic 6N and 6J mice. **I**–**L**, **U**–**X** the absolute bladder weight (BLW), BLW to BW ratio, BLW to TL ratio, and BLW to TL^3^ ratio of 6-month DC 6N and 6J mice. Unpaired *t*-test. *p < 0.05, **p < 0.01, ***p < 0.001. m, month(s). Grey background indicates significant alteration between groups

Six months post-STZ injection, both diabetic 6N and 6J mice showed sustained increase in HbA1c (6N, non-diabetic group 5.22 ± 0.70, diabetic group 9.59 ± 1.07, *p* < 0.001; 6J, non-diabetic group 6.12 ± 1.36, diabetic group 10.1 ± 1.61, *p* < 0.01). At this time point, diabetic mice had reduced absolute HW (Fig. 4A and M; 6N, 18.51%, 6J, 22.27%), while both substrains showed increased absolute KW (Fig. 4E and Q; 6N, 18.22%, 6J, 17.71%) and BLW (Fig. 4I and U; 6N, 62.40%, 6J, 30.56%). Interestingly, 6N diabetic mice also exhibited a significant increase in HW/BW ratios (Fig. 4B), similar to KW/BW (Fig. 4F and R; 6N, 81.56%, 6J, 54.19%) and BLW/BW (Fig. 4J and V; 6N, 143.52%, 6J, 71.67%) ratios, whereas no changes in HW/BW ratios were observed in 6J mice. TL and TL^3^ remained robust standard parameters for assessing cardiomyopathy in both substrains. Organ weights standardized to BW, particularly TL or TL^3^, were equally elevated in both 6N and 6J substrains, confirming the utility of these parameters for assessing long-term diabetic complications.

These results reveal that 6J mice are more sensitive to STZ-induced type 1 diabetes and exhibit greater organ weight changes over six months than 6N mice. TL and TL^3^, rather than BW, appear to be equally suitable parameters for normalization.

### Organ weights correlate with BW and TL in certain conditions in non-diabetic mice

**Fig. 5:**
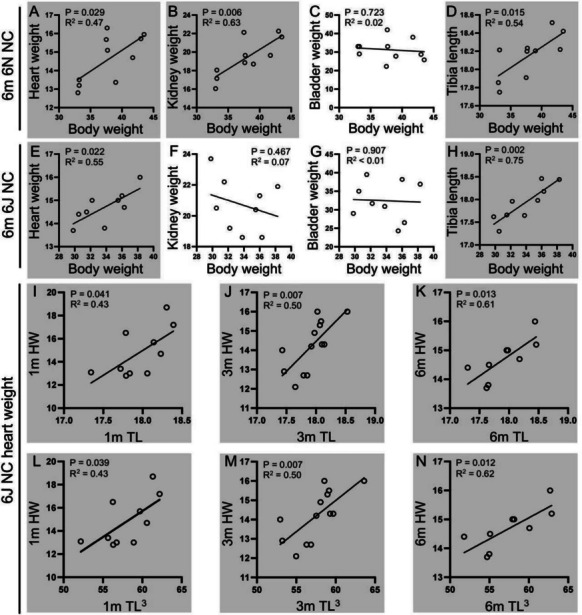
Organ weights correlate with BW and TL in certain conditions in non-diabetic mice. **A**–**H** scatter plots of the correlation of heart weight (HW, A, ⋅10 mg), kidney weight (KW, B, ⋅10 mg), bladder weight (BW, C, mg), and tibia length (TL, D, mm) with BW in 6-month 6N and 6J non-diabetic (NC) mice. **I**–**K** the correlation of HW with TL in 6N mice at 1, 3, and 6 months. **L**–**N** the correlation of HW with cubic tibia length (TL^3^) in 6N mice at 1, 3, and 6 months. m, month(s)

We further analyzed the correlation of organ weights with BW, TL, and TL^3^ (Fig. 5). Interestingly, a significant positive correlation was observed between HW, KW, and TL with BW in non-diabetic 6N mice at 6 months’ time point (Fig. 5A, B, and D), while the correlation between KW with BW was not demonstrated in 6J mice (Fig. 5F). This suggests that higher BW correlates with heavier HW and KW and longer TL, possibly due to age-related changes, as this correlation was not observed in 1- and 3-month time points in non-diabetic animals. However, this correlation was inconsistent in diabetic mice in either substrain (data not shown).

Moreover, HW was strongly correlated with TL and TL^3^ at all time points (1, 3, and 6 months; Fig. 5I to N), exclusively in non-diabetic 6J mice, suggesting that TL and TL^3^ are reliable standard parameters for assessing heart hypertrophy. In diabetic animals, these correlations were inconsistent across all 6N and 6J groups, supporting the previous conclusion that TL and TL^3^ are better suited than BW for assessing heart hypertrophy in 6J mice in studies of heart diseases.

## Discussion

In this study, we found that TL and TL^3^ are more reliable standardizing parameters than BW for normalizing not only cardiac hypertrophy but also KW and BLW across all time points in our diabetes trials. Additionally, we identified the 6J substrain, rather than the 6N, is more appropriate for studying early-stage diabetic complications in the kidneys and bladder, making it an ideal model for understanding the pathogenesis of such diseases.

A key finding of this study is the feasibility and reliability of using TL and TL^3^ to standardize organ hypertrophy in emaciated, STZ-induced diabetic mice. Although BW has traditionally been employed to normalize organ weights in rodent models of diabetes (D'Souza et al. 2011; Glastras et al. 2016; Nemoto et al. 2006), this method becomes unreliable in the context of diabetes-induced cachexia. Specifically, STZ-induced diabetic mice experience significant BW loss. As demonstrated in this study, normalizing organ weights to BW can artificially amplify the apparent hypertrophic response by inflating organ/BW ratios compared to absolute organ weights or ratios standardized to TL or TL^3^. TL has already been introduced in the context of cardiac diseases, including STZ-induced diabetic cardiomyopathy, to adjust cardiac mass and prevent inaccuracies due to weight loss. In contrast to the decreased BW, TL remained stable across all observed time points under diabetic conditions in both substrains. This finding aligns with previous studies that applied TL in cardiac hypertrophy models (Cao et al. 2015; De Blasio et al. 2020; Tate et al. 2019). Notably, STZ-induced diabetes resulted in a smaller heart size in our study, which is reflected in the HW/TL ratio but contrasts with the HW/BW ratio. This observation is consistent with several prior reports (Chandramouli et al. 2018; De Blasio et al. 2020; Trost et al. 2002; Tsai et al. 2018; Westermann et al. 2007). However, the effect of STZ-induced diabetes on HW remains controversial, as some studies have reported opposite findings, showing increased cardiac mass under similar conditions (Bernardo et al. 2022; Elmadbouh & Singla 2021; Huang et al. 2021). Therefore, these findings highlight the critical importance of selecting a stable and invariable standard for normalization. TL provides a physiologically relevant and diabetes-insensitive alternative to BW, thereby enhancing the accuracy of hypertrophy assessments in experimental diabetes research.

Qiunt et al. suggested that TL^3^, rather than TL, may be more appropriate for indexing cardiac volume or mass (Hagdorn et al. 2019). However, contrary to Qiunt et al.’s findings, TL^3^ offered no advantage over TL for standardization in our study. Our results demonstrated that TL^3^ is as reliable as TL for standardizing cardiac hypertrophy. Since TL^3^ was introduced to assess three-dimensional cardiac volume or mass, its potential benefits in evaluating left ventricle volume and mass in STZ-induced diabetic complication, rather than just the heart, requires further analysis. STZ-induced diabetic nephropathy and cystopathy have been extensively studied in C57BL substrains. While TL has been used as a standard for diabetic nephropathy, it has not been applied to diabetic bladder studies (Bivona et al. 2011; Yang et al. 2019). Our findings suggest that both TL and TL^3^ are reliable for assessing kidney and bladder hypertrophy, especially in the early stages of such diabetic complications.

Another important finding of our study is that the 6J substrain is more advantageous than the 6N for investigating early-stage diabetic complications, particularly diabetic cystopathy. Both 6N and 6J substrains are suitable for studying diabetic cardiomyopathy across all time points and the long-term diabetic complications. Previous studies have reported that 6J mice exhibit glucose intolerance, insulin resistance, and impaired insulin secretion due to a naturally occurring deletion of the Nicotinamide Nucleotide Transhydrogenase (Nnt) gene (Fergusson et al. 2014). Consequently, 6J mice are commonly used to model metabolic disorders. In our study, the 6J substrain demonstrated more pronounced hypertrophy related to diabetic complications than the 6N substrain, consistent with reported genetic differences (Simon et al. 2013). However, the sensitivity to early-stage diabetic complications appears to be substrain-dependent, whereas there was no difference between 6N and 6J mice in long-term diabetic models in our study. Our data showed that 6N mice were more sensitive to STZ treatment than 6J mice, suggesting that this sensitivity might be independent of the Nnt gene. STZ-induced diabetes models have shown varying responses to STZ depending on dosage, mouse strain, and gender (Ghasemi & Jeddi 2023; Goyal et al. 2016; Marino et al. 2023; Rashmi et al. 2023). In line with the 3R principle Replacement, Reduction, and Refinement in animal studies, wild-type mice were regularly refreshed through crossbreeding with the corresponding 6N or 6J backgrounds in this study. The higher sensitivity to STZ in 6N mice compared to 6J might be attributed to the strain differences in these backgrounds.

Both 6N and 6J substrains are the most commonly used animal models for studying cardiomyopathy. Despite the Nnt null mutation and possible impaired insulin secretion, 6J mice do not exhibit significant cardiac abnormalities up to 18 months of age. In contrast, 6N mice develop mild dilated cardiomyopathy from 12 months on, a phenotype attributed to a mutation in the myosin light chain kinase 3 (Mylk3) gene in the 6N substrain (J. L. Williams et al. 2020a, b). Their study found no differences in HW and HW/TL ratio between 6N and 6J mice. In our study, HW, HW/TL, and HW/TL^3^ ratios are comparable between these two non-diabetic substrains. Consistent with previous reports (Chandramouli et al. 2018; De Blasio et al. 2020; Trost et al. 2002; Tsai et al. 2018; Westermann et al. 2007), STZ-induced diabetes, triggered by a single high dose of STZ, resulted in reduced HW, HW/TL, and HW/TL^3^ ratios at 1, 3, and 6 months of post-diabetes induction, suggesting that STZ-induced diabetic cardiomyopathy is independent of the Mylk3 mutation. Another possibility is that the smaller heart size observed in STZ-induced cardiomyopathy represents an atrophic rather than dilated form of heart disease.

Our study demonstrated positive correlations between BW to HW and KW, and to TL assessed in non-diabetic 6N mice, whereas in the 6J mice, only heart and TL exhibited positive correlations with BW. These findings highlight strain-dependent physiological responses with significant pharmacological implications including organ scaling, substrain selection, and refinement of animal model design. Such positive correlations have been reported in men and C57BL mice under physiological conditions (Hagdorn et al. 2019; Mubbunu et al. 2018). This presents a substantial challenge, complicating data interpretation when body and organ weights do not alter simultaneously under pathological conditions, such as STZ-induced diabetes, or in toxicological research assessing the effect of chemical treatment (Bailey et al. 2004). Our observations suggest that the uncorrelated organs in 6J mice, such as the kidney, may be more susceptible to multifactorial impairments than those in 6N. This susceptibility may be attributed to genetic variation, social hierarchy among conspecifics, or generally small sample size in animal studies. Consistent correlation between HW and TL in control mice supports the appropriateness of TL as a standard in cardiac research, which has been revealed by reports in the field (Cao et al. 2015; De Blasio et al. 2020; Tate et al. 2019). However, this correlation was absent in our diabetic animals, suggesting that the diabetic condition, in which HW is disproportionately altered while TL remains unchanged, disrupted the correlation between HW and TL.

Nevertheless, it is important to emphasize that evaluating organ hypertrophy by standardizing organ to the TL or TL^3^ ratio is only an initial step. One of the limitations of the current study is the lack of morphological and molecular evidence, which is essential for the comprehensive characterization of organ hypertrophy. As part of our future work, organ function analysis, histopathological assessment, and investigations into the cellular and molecular mechanisms need to be conducted to draw more definitive conclusions regarding organ hypertrophy.

In conclusion, although the 6N substrain is more sensitive to STZ than the 6J, the 6J substrain serves as an excellent model for studying multiple diabetes-related complications, including diabetic cardiomyopathy, nephropathy, and cystopathy, especially in the early stage of the diseases. This model is highly valuable for exploring the progression and potential treatments for these diseases. Furthermore, when standardizing organ mass in experimental settings, TL offers a more accurate and reliable metric than BW, ensuring greater consistency in assessing diabetes-related organ hypertrophy.

## Supplementary Information

Below is the link to the electronic supplementary material.

ESM1 Supplementary Table 1. Tibia length of diabetic and non-diabetic mice among two substrains. (JPG 400 KB) NC: non-diabetic mice; DC: diabetic mice; Tibia length (mm) is presented as Mean ± SD

## Data Availability

The datasets used and/or analyzed during the current study are available from the corresponding author on reasonable request.

## Abbreviations

BG: Blood glucose
BLW: Bladder weight
BW: Body weight
DC: Diabetic condition
HbA1c: Hemoglobin A1C
HW: Heart weight
IDF: International Diabetes Federation
KW: Kidney weight
Mylk3: Myosin light chain kinase 3
NC: Non-diabetic condition
Nnt: Nicotinamide Nucleotide Transhydrogenase
STZ: Streptozotocin
TL: Tibia length
TL^3^: Cubic tibia length
6N: C57BL/6N
6J: C57BL/6J
