# Supplementary figures and images for: Tibia length is an appropriate standard for evaluating hypertrophy in streptozotocin-induced diabetic complications
Source: Naunyn Schmiedebergs Arch Pharmacol. 2025 Jul 17;399(1):695–707. doi: 10.1007/s00210-025-04413-3 (PMC12894438; doi:10.1007/s00210-025-04413-3)

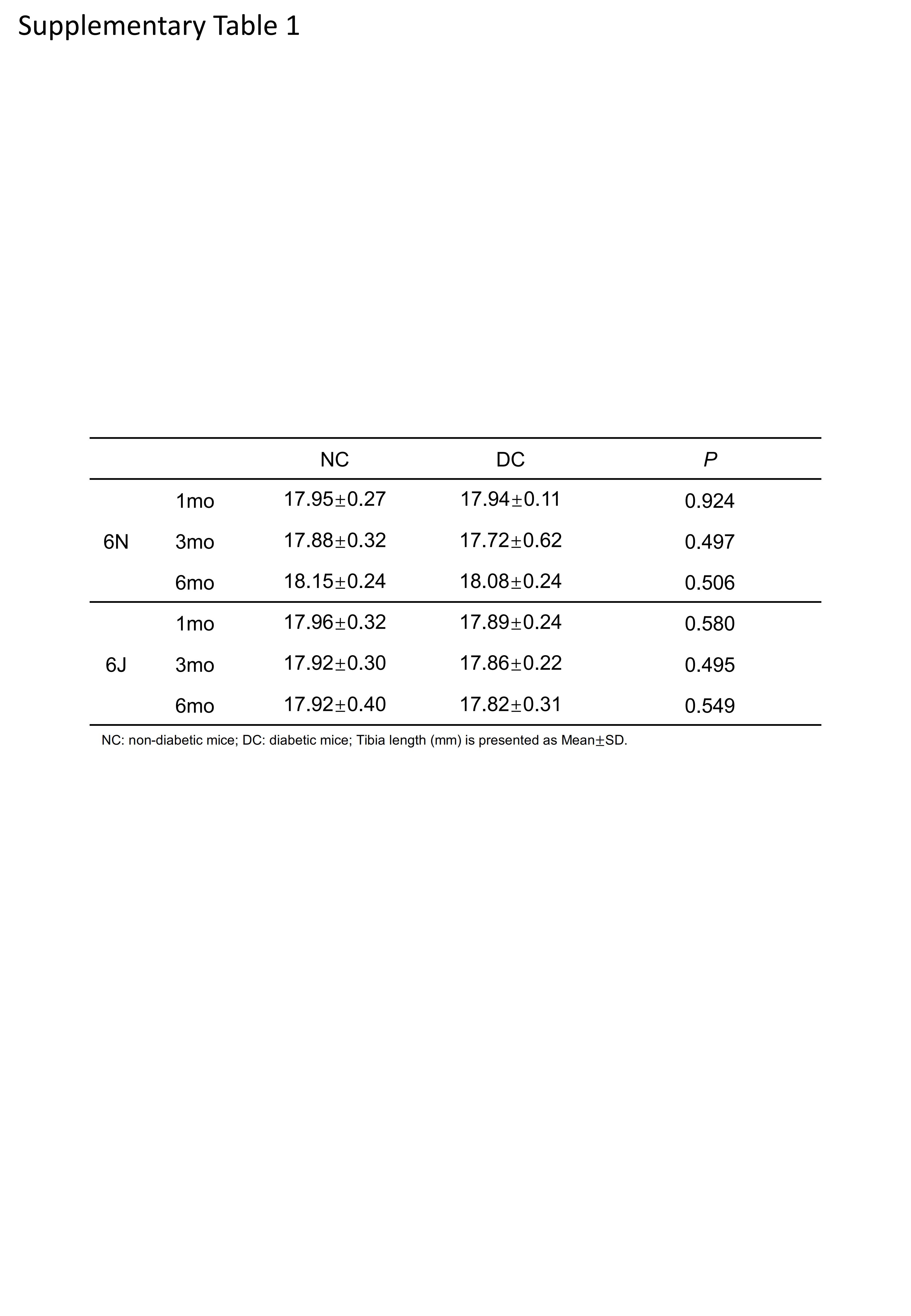

Supplement: Supplementary file 1 — Supplementary Table 1. Tibia length of diabetic and non-diabetic mice among two substrains. (JPG 400 KB) NC: non-diabetic mice; DC: diabetic mice; Tibia length (mm) is presented as Mean ± SD [file 210_2025_4413_MOESM1_ESM.jpg]
